# On Diseases of the Pancreas

**Published:** 1891-03-07

**Authors:** 


### ON DISEASES OF THE PANCREAS.

Some years ago Dr. F. Roberts found it necessary to insist on the importance of remembering that there is such an organ as the pancreas, and though attention has of late been directed to it from one or two quarters, the warning is still needed. We are just beginning to estimate by exact methods the importance of the changes in the gastric juice in disease, and to learn when and how to supply its defects, but hitherto we have little certain knowledge of changes in the pancreatic secretion. Every symptom has been found untrustworthy when taken alone, to prove the existence of pancreatic disease. Rapid and general emaciation was once relied on, but it follows disease of the other digestive organs when the pancreas is sound, and has not been found in many cases where the pancreas is atrophied or has occluded ducts whether in men or in animals operated on for the purpose. Similarly no form of ptyalism or diarrhœa has proved reliable. The presence of fat in the stools does seem to occur more often in pancreatic disease than in the affections of other organs, yet it may occur in perfect health from special diet, and in simple disease of the liver. The presence of unaltered muscle fibres has also a considerable diagnostic value. Jaundice and a special form of diabetes may at times be pancreatic in origin, but in the absence of reliable symptoms or methods of examination there can rarely be certainty in diagnosis. In one way we can sometimes get evidence of practical value. If, as Dr. F. Roberts pointed out, we find the symptoms disappear when pancreatised preparations are given, there is a strong presumption that the pancreas was at fault. The fact is the digestive organs do largely supplement each other's deficiencies. This has been recently confirmed by the important series of experiments carried out by Minkowski at Dorpat and Strasburg. He claims that no one previous to himself had succeeded in totally removing the pancreas and in yet keeping the animal alive except in the case of certain birds; nor does he think that the solar plexus was injured in his cases. This total extirpation then was followed in the first place by non-absorption of 56 per cent. of proteid foods, of only some 30 per cent. of the starch, and of all the fat except such as was contained naturally emulsified in milk. In the normal state of things the pancreas produces the absorption of nearly all the starch, yet here the intestinal tract made shift in its absence to take up 70 per cent. We were, indeed, aware that symptoms of pancreatic disease are uncertain because imperfect digestion of starches, proteids, or fats may individually depend on disease of the salivary glands, of the stomach, or of the liver, as well as on failure of the pancreas, but we could hardly expect such a degree of compensation as this. It was formerly thought that the one function performed by the pancreas alone was that of splitting up fats into fatty acids and glycerine, though recently it had been shown that a little was split up in the stomach. Minkowski now finds *all* the fats were so split up in the absence of the pancreas. Fat absorption failed indeed, but this seems to be contradicted in so many other cases that on this point further investigation seems needed. We are tempted to ask whether complete failure to digest starch, for example, does not imply disease of two organs? There is a physiological instance of this in the inability of either the pancreas or the salivary glands to change starch in the first months of life. Here neither organ by itself has any power of sugar formation, and there is complete failure of such digestion. Vittorio has recently shown that in many wasting diseases the saliva loses its sugar-forming power. Does the extreme wasting occasionally seen in pancreatic disease depend on concurrent affections of the pancreas and salivary gland?

The most interesting part of Minkowski's observations concerned the production of diabetes. The coincidence of diabetes and pancreatic disease has been noted in various cases since 1788, but the evidence seemed to point rather to the solar plexus as the real seat of the mischief. Thus Munk and Klebs found they could cause diabetes by partial extirpation of the plexus, while removal of the pancreas or occlusion of its ducts gave no results. Minkowski was able, with antiseptic precautions, to remove the gland completely without injuring the solar plexus, and found that diabetes of a severe form followed invariably. His results have been confirmed by several other persons. Two of these observers notice the close correlation of the pancreas with the salivary and other digestive glands, for they found that diabetes was often brought on, not only by extirpation of the pancreas, but even by removal of the salivary glands or of the duodenum. They imagine that in each of these organs a special ferment is present concerned with the destruction of sugar. The most important point probably in all these recent experiments is that no diabetes occurs if a moderate portion of the pancreas is left in the body, although it be quite cut off from the bowel. In other words, quite apart from its function of pouring into the bowel its special juice, it exercises some influence on the blood or lymph which flows through it, comparable to that of the thyroid and the ductless glands. Lepine mentioned at Berlin that he had drawn and set aside blood from a healthy dog and also from one deprived entirely of the pancreas, and found that not only was there twice as much sugar in the blood of the latter compared with that of the healthy dog, but that a rapid destruction of the sugar went on in the normal blood, while in 15 hours hardly any was lost from that of the mutilated animal. Clearly there was present in the blood some sugar-destroying ferment, which comes partly from the pancreas even when its ducts are occluded. We are not here concerned with the question as to the existence of the various forms of diabetes. It is abundantly clear that it is produced by affections of different parts of the body, and the symptoms are probably caused in very different ways. On the other hand, it is clear that the pancreas is responsible for certain severe cases of the disease, and there is reason to put these down to the failure of a sort of ferment produced by it. Unfortunately no indications for treatment have been offered except by Lepine, who carried out his theory by injecting a diastatic ferment into the blood of a diabetic patient, with the result that a great diminution of the glycosuria took place.

